# Exploring the glycosylation of mucins by use of O-glycodomain reporters recombinantly expressed in glycoengineered HEK293 cells

**DOI:** 10.1016/j.jbc.2022.101784

**Published:** 2022-03-02

**Authors:** Andriana Konstantinidi, Rebecca Nason, Tomislav Čaval, Lingbo Sun, Daniel M. Sørensen, Sanae Furukawa, Zilu Ye, Renaud Vincentelli, Yoshiki Narimatsu, Sergey Y. Vakhrushev, Henrik Clausen

**Affiliations:** 1 Copenhagen Center for Glycomics, Departments of Cellular and Molecular Medicine, Faculty of Health Sciences, University of Copenhagen, Copenhagen, Denmark; 2 Architecture et Fonction des Macromolécules Biologiques, CNRS, Aix-Marseille Université, Marseille, France; 3 GlycoDisplay ApS, Copenhagen, Denmark

**Keywords:** mucins, O-glycodomains, glycomucinases, cell-based glycan array, glycoengineering, glycosylation, glycosyltransferases, intact mass, ACN, acetonitrile, AOSM, asialo-OSM, FA, formic acid, GALNT, GalNAc transferase, HEK293, human embryonic kidney 293 cell line, KI, knockin, KO, knock out, MS, mass spectrometry, OSM, ovine submaxillary mucin, PSGL-1, P-selectin-glycoprotein ligand 1, SDC3, syndecan-3, TR, tandem repeat, VVA, villosa agglutinin

## Abstract

Mucins and glycoproteins with mucin-like regions contain densely O-glycosylated domains often found in tandem repeat (TR) sequences. These O-glycodomains have traditionally been difficult to characterize because of their resistance to proteolytic digestion, and knowledge of the precise positions of O-glycans is particularly limited for these regions. Here, we took advantage of a recently developed glycoengineered cell-based platform for the display and production of mucin TR reporters with custom-designed O-glycosylation to characterize O-glycodomains derived from mucins and mucin-like glycoproteins. We combined intact mass and bottom–up site-specific analysis for mapping O-glycosites in the mucins, MUC2, MUC20, MUC21, protein P-selectin-glycoprotein ligand 1, and proteoglycan syndecan-3. We found that all the potential Ser/Thr positions in these O-glycodomains were O-glycosylated when expressed in human embryonic kidney 293 SimpleCells (Tn-glycoform). Interestingly, we found that all potential Ser/Thr O-glycosites in TRs derived from secreted mucins and most glycosites from transmembrane mucins were almost fully occupied, whereas TRs from a subset of transmembrane mucins were less efficiently processed. We further used the mucin TR reporters to characterize cleavage sites of glycoproteases StcE (secreted protease of C1 esterase inhibitor from EHEC) and BT4244, revealing more restricted substrate specificities than previously reported. Finally, we conducted a bottom–up analysis of isolated ovine submaxillary mucin, which supported our findings that mucin TRs in general are efficiently O-glycosylated at all potential glycosites. This study provides insight into O-glycosylation of mucins and mucin-like domains, and the strategies developed open the field for wider analysis of native mucins.

Mucin-type (GalNAc-type) O-glycosylation is an abundant type of protein glycosylation initiated in the Golgi by a large family of up to 20 polypeptide GalNAc transferase (GALNT) isoenzymes with different kinetic properties and substrate specificities (1, 2). The repertoire of the GALNTs expressed in cells vary, and GalNAc-type glycosylation (hereafter, simply O-glycosylation) is therefore uniquely suited to differentially regulate the positions in proteins being glycosylated in cells (3). O-glycans are found on select Ser and Thr residues (and Tyr) often in clustered motifs with adjacent Pro residues, but no simple consensus sequence motifs have emerged (1, 2, 4, 5). Prediction algorithms for O-glycosylation such as NetOGlyc4.0 (http://www.cbs.dtu.dk/services/NetOGlyc-4.0/) (5) and GALNT isoform specific (IsoGlyP) (6) provide valuable tools. Advances in O-glycoproteomics employing genetic engineering for simplification of glycan structural heterogeneity (SimpleCells) (5, 7), improved and novel enrichment strategies (8, 9, 10, 11), and enhanced sensitivity and speed of mass spectrometry (MS) (12) have advanced insights into O-glycosites in around 3000 human proteins trafficking the secretory pathway (13, 14). Paradoxically, the classes of proteins predicted to be the most heavily O-glycosylated, that is, mucins and glycoproteins with mucin-like domains comprised of high frequencies of Ser/Thr residues, are those with the least experimental evidence to support the positions where O-glycans are attached (14, 15, 16, 17, 18, 19, 20, 21). This conundrum is likely primarily a result of available experimental strategies, where the main obstacle is limited options for proteolytic digestion of O-glycodomains into fragments suitable for MS sequencing because of a characteristic amino acid usage in the domains, generally without charged residues, and the high density of O-glycans (14, 15). Regardless, considerable experimental data for O-glycan positions exist for the human cell membrane mucin, MUC1 (22), the mucin-like lubricin/proteoglycan 4 (23), and fragments of the porcine and canine submaxillary mucins (17, 18, 19, 21).

At least 18 distinct human genes encode membrane or secreted mucins (24), and a larger number of genes encode proteins with mucin-like domains. Most mucins contain large O-glycodomains with variable number of more or less conserved tandem repeat (TR) sequences (25, 26, 27), whereas O-glycodomains found in other proteins mainly do not. The classification of mucins may in this respect not be consistent, for example, the P-selectin-glycoprotein ligand 1 (PSGL-1) contains an O-glycodomain with characteristic TRs (28), whereas the cell membrane glycoprotein classified as MUC16 (CA125) has a very large N-terminal O-glycodomain without apparent TRs (29, 30, 31). Importantly, TRs in mucins are quite distinct in length and sequence with characteristic spacing of potential Ser/Thr O-glycosites, and these features diverge among closely related mammals (25, 32). We and others have proposed that mucin TRs as well as other O-glycodomains contain unique codes formed by O-glycan clusters and/or patterns that serve as recognition motifs for receptors (33, 34). In order to explore this, we recently developed a cell-based mucin TR array platform to display and produce small fragments (around 200 amino acids) of O-glycodomains derived from the characteristic TR regions (34). This platform relies on a library of stable gene-engineered human embryonic kidney 293 (HEK293) cells with distinct O-glycosylation capacities and expression of a panel of recombinant GFP-tagged mucin TR reporters either as cell membrane retained or secreted proteins. The display of mucin TRs on the cell surface provided a tool to demonstrate that human Siglecs and microbial Siglec-like adhesins appear to recognize their cognate O-glycan ligands with high selectivity for their presentation on mucin TRs (34, 35, 36), indicating that it is important to identify the actual O-glycan sites in mucin TRs. We therefore used expression of secreted mucin TR reporters to start characterizing O-glycosylation, and interestingly, we were able to analyze the entire O-glycodomains of several mucin TR reporters with the simplest glycoform (Tn, GalNAcα1-O-Ser/Thr) by intact MS and demonstrate that most of the mucin reporters were O-glycosylated with exceptionally high fidelity and near complete occupancy of all potential Ser/Thr O-glycosites (34).

Intact MS analysis of glycoproteins has primarily been applied to abundantly available recombinant N-glycoprotein therapeutics including immunoglobulin G and erythropoietin (37, 38, 39). More recently, intact MS analysis was applied for direct profiling of human plasma N-glycoproteins, enabling insight into disease states (40, 41) as well as glycoprotein–drug (42) and/or glycoprotein–lectin (43) interactions. Heterogeneity in glycan structures attached to proteins constitutes the main obstacle for use of intact MS for analysis, and this is where genetic glycoengineering can be applied to obtain more homogenous glycoproteoforms (39, 44). Intact MS may be particularly suited for O-glycoproteins with dense O-glycodomains that are poorly accessible to commonly used bottom–up proteomics workflows (34), since intact MS can provide a holistic picture of intact O-glycoprotein macroheterogeneity where the total sum of attached O-glycans may be estimated. One illustrative recent example is the therapeutic chimeric tumor necrosis factor alpha receptor fusion protein (Etanercept) with multiple N-glycans and up to 26 O-glycans attached, where intact MS after removal of N-glycans was used to estimate O-glycan occupancy revealing the presence of 14 to 23 core1 O-glycans (38). However, because of the known issues of mass degeneracy between extended O-glycans and different O-glycan cores, it is very challenging to profile O-glycoproteins at both the microheterogeneity and macroheterogeneity levels if the glycoprotein in question carries more than one type of O-glycan structure (45).

Here, we extended our previous studies of mucin O-glycodomains to include a more comprehensive panel of mucin TR reporters derived from secreted and cell membrane human mucins as well as examples of mucin-like O-glycodomains. When possible, we combined intact MS analysis of excised O-glycodomain reporters with bottom–up analysis to characterize sites of O-glycosylation. Bottom–up site-specific analysis was performed with both select peptidases (Glu-C, trypsin, and Asp-N) as well as glycomucinases (StcE [secreted protease of C1 esterase inhibitor from EHEC], BT4244) (16, 46, 47), which provided further insights into the substrate specificities of bacterial glycoproteases. We begin to address regulation of sites of O-glycosylation in mucin TR domains by the repertoire of GALNTs as well as the elongation process and discovered that the elongation of O-glycans with the core3 structure adversely affects the O-glycan occupancy. Finally, we used the peptidases and glycomucinases for bottom–up analysis of ovine submaxillary mucin (OSM), which led to unambiguous identification of the gene and full coding sequence from a recent genome draft.

## Results

**Figure 1:**
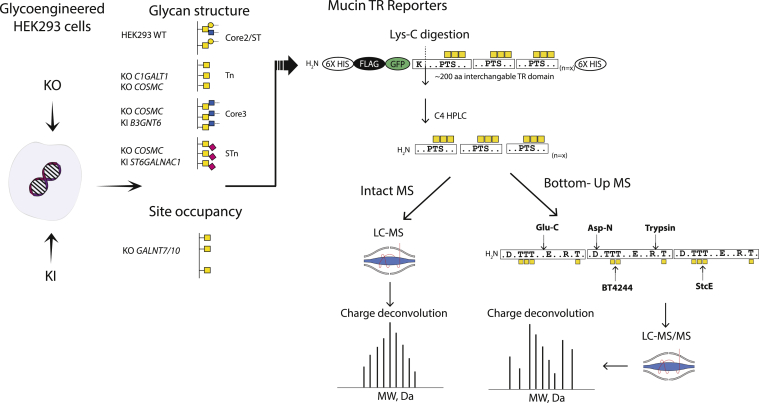
**Overview of the cell-based production of mucinTRs and the analytic workflow.** Mucin TR reporters were expressed in stably glycoengineered HEK293 cells to produce defined O-glycoforms, including Tn (KO *C1GALT1*), mSTa (KO *GCNT1/ST6GALNAC2/3/4*), and core3 (KO *COSMC*/KI B3GNT6) O-glycosylation, and to produce glycoforms with different O-glycan occupancy (KO *GALNT4*; KO *GALNT7*/*10*) (*left panel*). The secreted mucin reporters contain N-terminal GFP, multiple tags (6×His, FLAG tags), and the interchangeable mucin TR O-glycodomains (approximately 200 amino acids). Digestion with Lys-C results in release of the intact O-glycodomain without GFP (except with rare O-glycodomains containing internal Lys residues). This enables intact MS of the isolated O-glycodomains and/or bottom–up analysis after digestion with Glu-C, Asp-N, and trypsin (*right panel*). Glycan symbols are drawn according to the SNFG nomenclature (94). HEK293, human embryonic kidney 293 cell line; MS, mass spectrometry; SNFG, Symbol Nomenclature for Glycans; TR, tandem repeat.

The cell-based platform for production of mucin O-glycodomains with rather homogenous O-glycans opens up for detailed structural analysis as outlined in Figure 1 (34, 35). We designed secreted reporters containing TR O-glycodomains derived from a panel of secreted and membrane-bound human mucins as well as mucin-like domains from glycoproteins and expressed these in glycoengineered HEK293 isogenic cells selected to produce different O-glycan structures and densities of O-glycans. The glycoengineered cell lines used were developed previously, and for all genetic glycoengineering designs, multiple clones (usually two to four) were generated and characterized (35). Here, we used one representative clone for expression of the mucin reporters and subsequent analysis. We aimed for intact MS analysis of the isolated O-glycodomains and, when possible and relevant, bottom–up analysis to support interpretation of O-glycan occupancy and identification of O-glycosites (Fig. 1). The design of reporters in most cases enabled release of the C-terminal O-glycodomain by Lys-C digestion and following purification by reverse-phase (C4) HPLC direct analysis by intact MS. For several reporters, we were also able to perform bottom–up analysis by digestion with proteases (Glu-C, trypsin, and Asp-N) and/or glycomucinases (BT4244 and StcE).

### Intact MS analysis of mucin TRs and mucin-like O-glycodomains carrying truncated Tn O-glycans

We used HEK293 SimpleCells with KO of either *COSMC* (HEK293^KO^ ^*COSMC*^) or *C1GALT1* (HEK293^KO^ ^*C1GALT1*^) encoding the private chaperone for the core1 synthase or the synthase itself, respectively, resulting in mucin TR reporters with only the simplest O-glycan structure, GalNAcα1-O-Ser/Thr, also designated Tn. We previously reported intact MS analysis of representative TR domains derived from several secreted (MUC2, MUC5AC, and MUC7) and transmembrane (MUC13 and MUC22) human mucins by the use of mucin TR reporters recombinantly expressed in glycoengineered HEK293 cells (34). Intact MS analysis of these revealed that most, if not all, of the 50 to 100 potential Ser/Thr glycosites were glycosylated (Table 1).

**Table 1:** Overview of intact MS analysis of mucin TR reporters expressed in HEK293^KO*C1GALT1*^

Tn-mucin TR reporters	Representative mucin TR sequence and number of TRs included in reporter	Pot O-glycosites in TR reporters	Three most abundant HexNAc proteoforms identified^a^	Full range of HexNAc proteoforms identified^a^	Averaged HexNAc incorporated per TR^b^ (exp/pot)
MUC2 TR1^c^	P**S**PPI**TTTTT**PPP**TTT**_10x_	86	79–81	73–86	9/9
MUC2 TR2^c^	G**T**Q**T**P**T**P**T**PI**TTTTT**V**T**P**T**P**T**P**T**_7x_	89	87–89	85–90	13/13
MUC5AC^c^	**STTS**AP**TT**_18x_	103	101–103	98–105	6/6
MUC7^c^	**TT**AVPP**T**P**S**A**TT**LDP**SS**A**S**APPE_7x_	67	62–64	58–67	9/9
MUC1^c^	APD**T**RPAPG**ST**APPAHGV**TS**_7x_	34	32–34	28–35	5/5
MUC4	PLPV**T**D**TSS**A**ST**GHA**T**_9x_	65	60–62	49–66	7/7
MUC13^c^	**TS**DII**T**A**SS**PNDGLI**T**_9x_	58	51–53	48–55	6/6
MUC17	**TST**P**S**EG**ST**PF**TS**MPV**ST**MPVV**TS**EA**ST**_5x_	73	62–64	42–70	13/14
MUC20	**S**E**SS**A**SS**DGPHPVI**T**P**S**RA_8x_	56	26–28	10–42	3/7
MUC21	**SS**GA**ST**A**T**N**S**E**SST**V_10x_	91	45–47	30–65	5/9
MUC22^c^	**S**E**TT**V**TST**AG_15x_	83	68–71	59–75	5/6
SDC3	d	52	48–50	45–52	d
PSGL-1	Q**TT**QPAA**T**EA_14x_	47	38–40	37–40	3/3

a HexNAc residues identified by intact MS.

b Comparison of the experimental (exp) number of HexNAc residues identified and the number of potential (pot) O-glycosites per TR. The values are averaged (Avr) numbers based on most abundant number of HexNAc residues and the number of Ser/Thr O-glycosites available in the most common imperfect TR sequence.

c Previously reported (34).

d SDC3 does not contain TRs.

**Figure 2:**
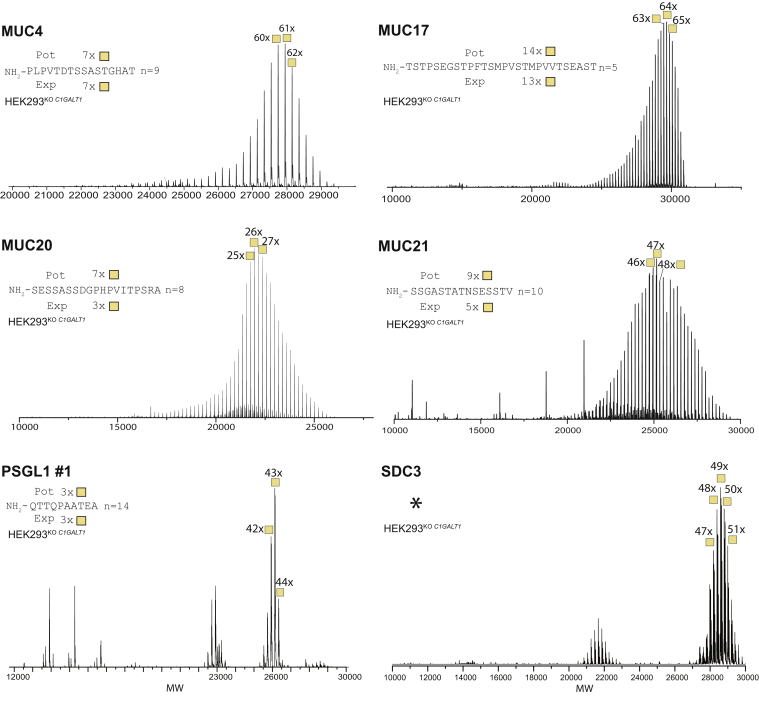
**Intact MS analysis of O-glycodomains isolated frommucinTR reporters with Tn O-glycans.** Deconvoluted intact mass spectra of isolated O-glycodomains from MUC4, MUC17, MUC20, MUC21, PSGL-1, and SDC3 mucin TR reporters expressed in HEK293^KO^^*C1GALT1*^ cells. The three most abundant masses are annotated with the predicted number of attached HexNAc residues. A representative TR sequence is shown with the number of total potential (Pot) O-glycosylation sites (Ser/Thr residues) and the experimentally (Exp) predicted average number of HexNAc residues per TR. SDC3 does not contain TRs, and the full sequence of the mucin-like domain is shown in Fig. S1. HEK293, human embryonic kidney 293 cell line; MS, mass spectrometry; PSGL-1, P-selectin-glycoprotein ligand 1; SDC3, syndecan-3; TR, tandem repeat.

Here, we expanded the intact MS analysis to TRs derived from additional transmembrane mucins and O-glycoproteins with mucin-like domains (Fig. 2 and Table 1). The isolation and intact MS protocols were developed with the Tn-MUC1 glycodomain and demonstrated to yield reproducible results (34), and the intact MS analyses presented were in general performed once. Interestingly, the Tn O-glycan occupancy of select transmembrane mucins was considerably lower than the available Ser/Thr glycosites. In particular, the predominant glycoforms of MUC20 and MUC21 were predicted to have ∼50% occupancy. The TR reporters for the transmembrane mucins, MUC1, MUC4, and the mucin-like reporters, PSGL-1 and syndecan-3 (SDC3), were predicted to have close to 100% occupancy, and the predominant glycoforms for MUC17 and MUC22 were predicted to have only slightly lower occupancy than the available total potential glycosites. Thus, the substantially lower O-glycan occupancy found for MUC20 and MUC21 does not seem to relate to the expression of TRs from membrane-bound mucins in secreted TR reporters, although it should be noted that one study has shown differences in O-glycan processing of the membrane-bound mucin, MUC1, when expressed as a transmembrane protein compared with when expressed as a truncated secreted protein (48). More likely, the lower occupancy for the MUC20 and MUC21 TR domains is related to a greater diversity in amino acid usage and a higher use of Ser than Thr residues in these TRs compared with what is found in secreted mucin TRs. The sequence context of O-glycosites clearly affects O-glycosylation, and Ser O-glycosites are considered poorer acceptor substrates than Thr sites for GALNTs (49), and GalNAc residues attached to Ser and Thr residues attain distinct conformations (50). We are currently not able to analyze TR reporters expressed as membrane-bound proteins.

The TR reporter designs for several mucin O-glycodomains also included potential isolated N-glycosylation sites (MUC7, MUC13, MUC17, and MUC22) (Fig. S1). N-glycosylation consensus sites (NXS/T) are occasionally found in mucin TRs and in particular in cell membrane mucins and mucin-like domains, but whether these are utilized has not been evaluated to our knowledge. The MUC22 TR reporter includes the N-glycan sequon -ETTTNSTTSSE- which provided an opportunity to analyze this (Fig. S2). Intact MS analysis following PNGaseF treatment revealed a characteristic mass shift of ∼2500 Da for all major glycoforms leaving a minor group of glycoforms (*m/z* 32,250–34,000) unchanged (Fig. S2), indicating that most of the TR glycoforms indeed contained a complex-type N-glycan.

### Intact MS analysis to probe the effects of the GALNT repertoire on O-glycan occupancy

The initiation step of O-glycosylation is controlled by multiple GALNTs with distinct and partly overlapping acceptor substrate preferences and kinetic properties, and the repertoire of expressed GALNTs in cells vary although several isoenzymes including GALNT1 and GALNT2 are rather ubiquitously expressed (1). Recent *in vitro* and *in cell* studies of the acceptor substrate specificities of GALNTs with detectable activity have demonstrated that these have considerable overlapping functions and only limited nonredundant substrate sites (4, 51, 52, 53). For example, analysis of HEK293 cells with individual KO of *GALNT1*, *T2*, *T3*, *T4*, *T7*, and *T10* genes revealed that the nonredundant contributions of these isoenzymes to the cellular O-glycoproteome are quite limited (51). Studies of O-glycosylation of mucin TRs are mainly limited to *in vitro* studies with peptide substrates (54), and these indicate that the typical Pro-Thr-Ser-rich sequences are broad substrates for most GALNTs (1, 6, 55). The so-called follow-up GALNTs (GALNT4, T7, T10, T12, and T17) selectively serve prior GalNAc-glycosylated substrates (56, 57, 58, 59, 60), and it is predicted that these are especially important for mucin TR substrates with dense and clustered glycosites (51). Previously, we showed that the close paralogs GALNT7/10 are important for glycosylation of dense O-glycodomains (51, 61), and we therefore predicted that dissection of the function of these would be particularly interesting with the mucin TR reporters.

**Figure 3:**
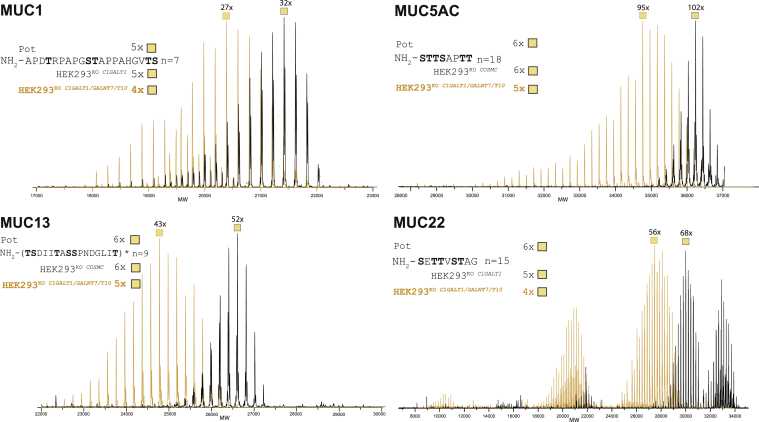
**Intact MS analysis of O-glycodomains isolated frommucinTR reporters with altered O-glycan occupancy.** Overlay of deconvoluted intact mass spectra of mucin TR reporters produced either in HEK293^KO^^*C1GALT1*^ or in HEK293^KO^^*COSMC*^ (*black contour*) and in HEK293^KO^^*C1GALT1*,*GALNT7/10*^ (*orange contour*). The most abundant masses are annotated with predicted number of HexNAc residues. A representative TR sequence is shown with indicated total potential glycosylated Ser/Thr residues (Pot) and the experimentally determined average number of HexNAcs found per each TR domain (HEK293^KO^^*C1GALT1 or COSMC*^, HEK293^KO^^*C1GALT1/GALNT7/T10*^). Relative abundances, deconvoluted masses, annotation, and theoretical masses of all peaks above 5% intensity are given in Supporting File S1. For MUC5AC and MUC13 TR reporters, we used the same raw files from previous work (34), but we modified the deconvolution parameters (see Data analysis in the Experimental procedures section). HEK293, human embryonic kidney 293 cell line; MS, mass spectrometry; TR, tandem repeat.

Here, we used intact MS to analyze O-glycans occupancy of mucin TR reporters expressed in HEK293^KO^ ^*C1GALT1*^ cells with additional double KO of *GALNT7* and *T10*. Four of the mucin TRs were not substantially affected by the loss of *GALNT7*/*T10* (Fig. S3), whereas the MUC1, MUC5AC, MUC13, and MUC22 TR reporters showed distinct shifts in mass range corresponding to loss of approximately one HexNAc residue per number of TRs included in reporters (Fig. 3). The lower number of HexNAcs incorporated in the latter TRs suggested that GALNT7 and T10 serve nonredundant functions. We chose to investigate the lower occupancy found for the MUC1 TRs, since this TR is quite conserved and amenable to Asp-N digestion and bottom–up LC–MS/MS analysis, as previously described (62). The GALNT follow-up process was originally discovered with the MUC1 TR substrate finding that only GALNT4 glycosylated two of the five glycosites (Ser in VTSA and Thr in PDTR) in the MUC1 TR and only following prior addition of GalNAc residues by other GALNTs (56, 63). However, we recently found that GALNT4 apparently only directs O-glycosylation at Thr in PDTR in HEK293 cells by analysis of the MUC1 TR reporter expressed in HEK293^KO^ ^*COSMC*/*GALNT4*^ cells (34). Since KO of *GALNT7/10* resulted in loss of six to seven GalNAc residues for the MUC1 reporter (approximately one per TR), we predicted that these isoforms selectively served the Ser residue in VTSA not glycosylated by GALNT4 in HEK293 cells. Surprisingly, loss of *GALNT7/T10* did not appear to affect any specific sites as all identified major species contained GalNAc residues at all five sites and all glycoforms contained GalNAc residues at Thr in PDTR and GSTA (Fig. S4). Since the TRs of MUC5AC, MUC13, and MUC22 are less conserved and without obvious digestion strategies for bottom–up analysis, we did not analyze these further.

Interestingly, we observed minor glycoproteoforms with a predicted number of HexNAc residues in excess of the total number of available potential Ser/Thr O-glycosites in the O-glycodomain reporters (Fig. 2), in agreement with our previous study (34). The basis for this is still unclear, but when the corresponding bottom–up analysis of the TR reporters was performed, this did not show evidence of excess HexNAc incorporation. One possible explanation may be a very minor incorporation of HexNAc_2_ disaccharides by the GALNTs themselves, which when accumulated over the 50 to 100 glycosites produces a small visible mass with a single excess HexNAc. The existence of GalNAcα1–3GalNAcα1-O-Ser/Thr O-glycans has been suggested in human meconium (64).

### Intact MS to probe the effect of O-glycan elongation on occupancy

**Figure 4:**
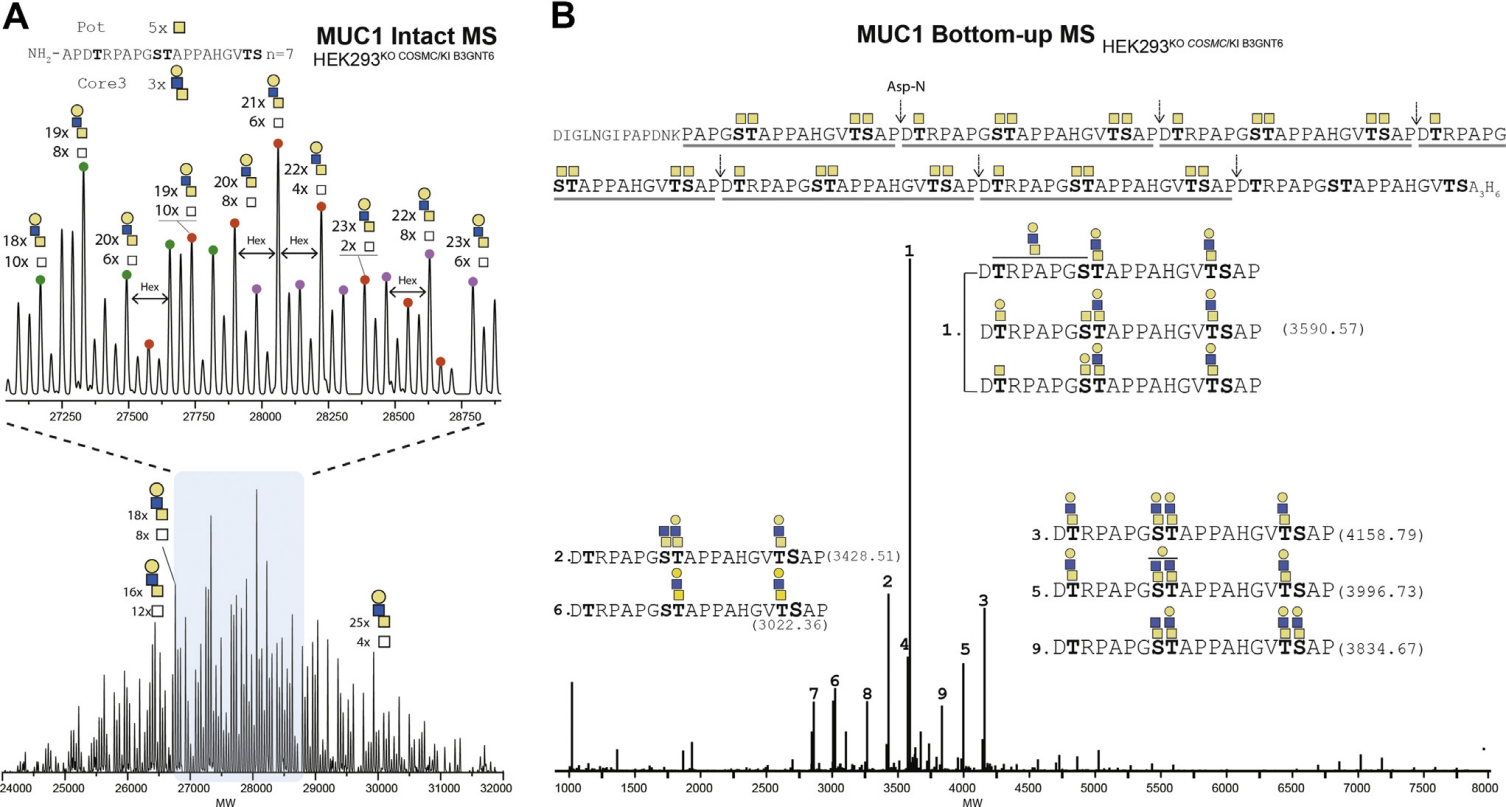
**Intact and bottom–up MS analysis of the isolated MUC1 O-glycodomain with core3 O-glycans.***A*, deconvoluted spectrum of intact MS analysis of the isolated TR O-glycodomain from the MUC1 reporter expressed in HEK293^KO^^*COSMC*, KI B3GNT6^. The most abundant masses are annotated with predicted number of HexHexNAc_2_ residues. A representative TR sequence is shown with indicated total potential (Pot) O-glycosylation sites (Ser/Thr residues) and the experimentally (Exp) observed average number of HexNAc residues per TR. *B*, deconvoluted spectrum of bottom–up analysis of the same MUC1 TR O-glycodomain with core3 O-glycans. The full MUC1 TR O-glycodomains sequence shows observed fragments (*underlined*) and Asp-N cleavage sites (*arrow*). *Bold* S/T *letters* represent unambiguously annotated glycosites (full sequence), and potential glycosites (peptides) and ambiguous sites are indicated by a *line*. The numbers assigned to each peak from one to nine are given based on decreasing abundance. Experimental masses in dalton for each assigned peak are provided in Supporting File S1. Only peaks with intensity above 10% were assigned. HEK293, human embryonic kidney 293 cell line; MS, mass spectrometry; TR, tandem repeat.

While intact MS analysis of the Tn-glycoforms of several mucin TR reporters was successful, we were unable to obtain interpretable results with more complex glycoforms including STn and T. The only exception was the MUC1 TR, although only after removal of sialic acids by neuraminidase treatment (34). Previously, we used intact MS analysis of the MUC1 TR reporter expressed in HEK293 cells with engineered glycosylation capacities limited to Tn, STn, T, or ST O-glycosylation to demonstrate that the core1 (T/ST) glycosylation pathway did not substantially affect the number of O-glycans attached (34). Interestingly, site-directed knockin (KI) of *ST6GALNAC1* in HEK293^KO^ ^*COSMC*^ cells to introduce STn also did not substantially affect initiation, in contrast to what was previously found with overexpression (65). Previous studies demonstrated that overexpression of ST6GALNAC1 in cell lines interferes with and overrides normal glycosylation leading to truncated STn O-glycans (65, 66), although potential effect on O-glycan occupancy was not investigated. Here, we were able to extend this to the core3 O-glycosylation pathway directed by B3GNT6 (67). B3GNT6 is only expressed in the normal gastrointestinal tract and downregulated in cancer, and interestingly, B3GNT6 is not expressed in common cancer cell lines (68). Expression of the MUC1 reporter in HEK293^KO^ ^*COSMC*,KI B3GNT6^ and analysis of the O-glycodomain by intact MS indicated a substantial reduction in the number of HexHexNAc_2_ O-glycans incorporated (Fig. 4*A*), predicted from the dominant 32 to 34 to 19 to 21 with a wider range of glycoproteoforms. This interpretation was confirmed by bottom–up analysis revealing rather homogeneous core3 O-glycan structures and demonstrating that the loss of total incorporated O-glycans was due to select loss of O-glycans at Ser in GVTS, Ser in GSTA, and partly Thr in PDTR (Figs. 4*B* and S5). Since these sites are predicted to be glycosylated through lectin-mediated properties of GALNTs (56, 69), this suggests that the core3 B3GNT6 synthase, in contrast to the core1 C1GALT1 synthase, competes with the GALNT follow-up reaction. The initiation and elongation of O-glycosylation takes place in common Golgi compartments, and the elongation process has the potential to compete and interfere with the initiation step orchestrated by GALNTs. Specifically, the initiation step by GALNTs involves so-called follow-up reactions where GALNTs utilize their lectin domains to bind prior incorporated GalNAc residues and efficiently complete glycosylation of adjacent glycosites, and premature elongation of these initial GalNAc residues is predicted to block lectin recognition and efficient glycosylation (69).

Interestingly, we found evidence of residual core1 O-glycans on the MUC1 reporter expressed in HEK293^KO^ ^*COSMC*,KI B3GNT6^ (Fig. 4*B*). We were unable to quantify the exact levels, but the core1 structure was found at Thr in PDTR and/or Ser in GSTA, and we also identified glycoforms with a single HexNAc (Tn) at these sites. Based on ELISA assays with lectins and antibodies, the majority of the O-glycans appears to represent core3 O-glycans since core1 (*Arachis hypogeae* agglutinin [PNA]) and Tn (*Vicia villosa* agglutinin [VVA]) were not or only barely detectable, respectively (34). While the ELISA results are semiquantitative, the results fully support the bottom–up MS analysis. The presence of minor levels of core1 structures is likely because of residual core1 C1GALT1 synthase activity, since the chaperone COSMC was KO for this experiment. We originally used KO of the *COSMC* gene to eliminate core1 elongation in cells (7), but we have noticed the presence of minor levels of residual core1 O-glycopeptides when using Jacalin enrichment of O-glycopeptides instead of the original VVA enrichment. The minor levels of core1 may be due to partial folding of the core1 synthase C1GALT1 in the absence of its private COSMC chaperone or the existence of other chaperones. To circumvent this issue, we have subsequently targeted the *C1GALT1* gene and used HEK293^KO^ ^*C1GALT1*^ cells.

The observed slightly incomplete galactosylation of the core3 disaccharide (HexNAc_2_) may be due to HEK293 cells only expressing B4GALTs and not B3GALTs, which may be a preferred pathway for core3 (35).

### Bottom–up MS of mucin TRs and O-glycodomains using Glu-C and trypsin

Most mucin TRs are not amenable for protease digestion and bottom–up MS analysis, but TRs in select transmembrane mucins, including MUC20 and MUC21, contain conserved Arg or Glu residues that may be digested by trypsin and Glu-C, respectively (Fig. 1). We therefore analyzed the MUC20 and MUC21 TR reporters expressed in HEK293^KO^ ^*C1GALT1*^ and predicted by the intact MS analysis to have low O-glycan occupancy (Fig. 2). Cumulatively, all potential Ser/Thr glycosites were found with an O-glycan in the identified glycopeptides; however, the individual glycopeptides identified contained GalNAc residues placed in different glycosite combinations, and there was no evidence of apparent preference for Thr or Ser glycosites (Fig. S6). Previous *in vitro* GALNT enzyme studies have demonstrated that the order of incorporation of GalNAc residues at different glycosites in peptides can affect the subsequent incorporations and hence generate different, mutually exclusive glycosylation patterns (70), but the basis for the lower occupancy observed is unknown.

**Figure 5:**
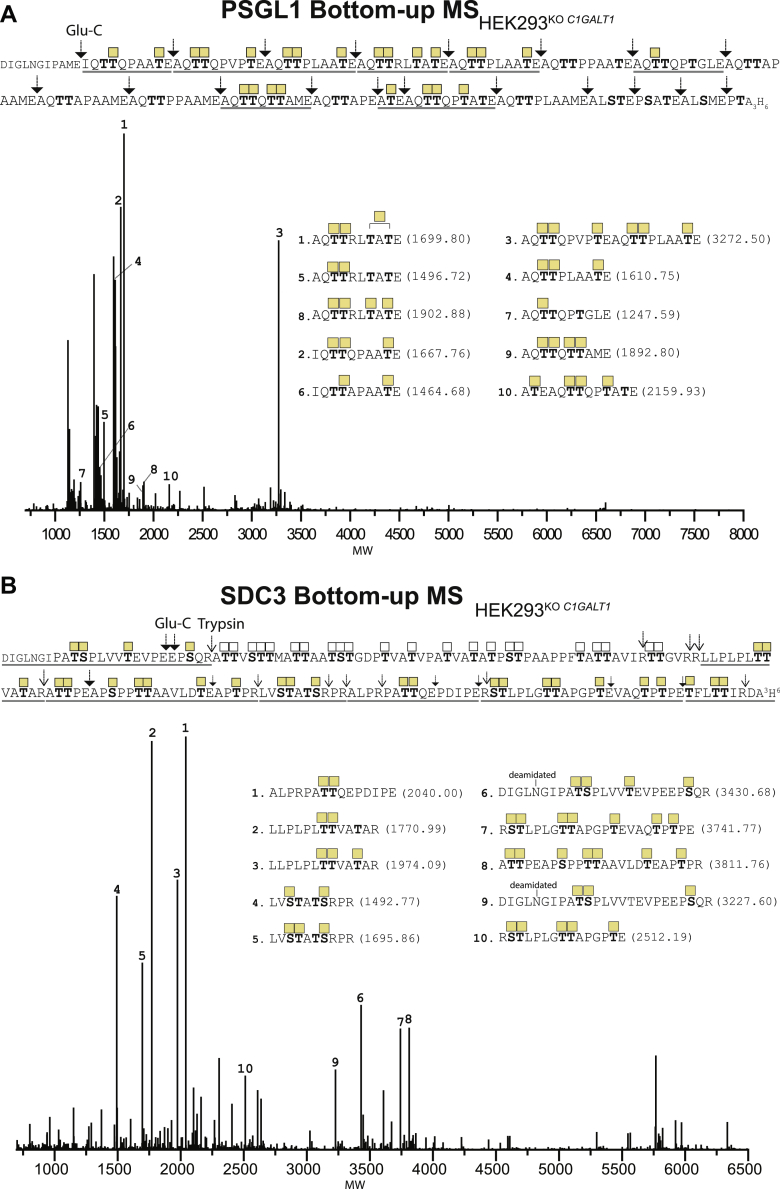
**Bottom–up analysis of O-glycodomains with Tn O-glycans using trypsin and Glu-C**. *A* and *B*, deconvoluted spectrum of bottom–up analysis of (*A*) PSGL-1 and (*B*) SDC3 isolated O-glycodomains from reporters expressed in HEK293^KO^^*C1GALT1*^. The numbers assigned to each peak from 1 to 10 are given based on decreasing abundance. Experimental masses in dalton for each assigned peak are provided in Supporting File S1. A 53 amino acid N-terminal sequence segment in SDC3 was only identified as a precursor ion without sequence and ETD verification of glycosites (*open squares*). Only peaks with intensity above 10% were assigned. ETD, electron-transfer dissociation; HEK293, human embryonic kidney 293 cell line; PSGL-1, P-selectin-glycoprotein ligand 1; SDC3, syndecan-3.

The TR O-glycodomain of PSGL-1 was also amenable for bottom–up analysis using Glu-C, and as shown from the intact MS analysis (Fig. 2), all potential glycosites were found to be occupied (Fig. 5*A*). We were also able to perform bottom–up analysis of SDC3 with sequential treatment of trypsin and Glu-C to demonstrate that essentially all glycosites were utilized (Fig. 5*B*). Interestingly, this correlates well with the current accumulated information of utilized O-glycosites in SDC3 as summarized in the GlycoDomainViewer (13) as well as with the results of the intact mass analysis of SDC3 (Fig. 2).

### Bottom–up MS analysis of mucin TRs using the glycomucinases StcE and BT4244

**Figure 6:**
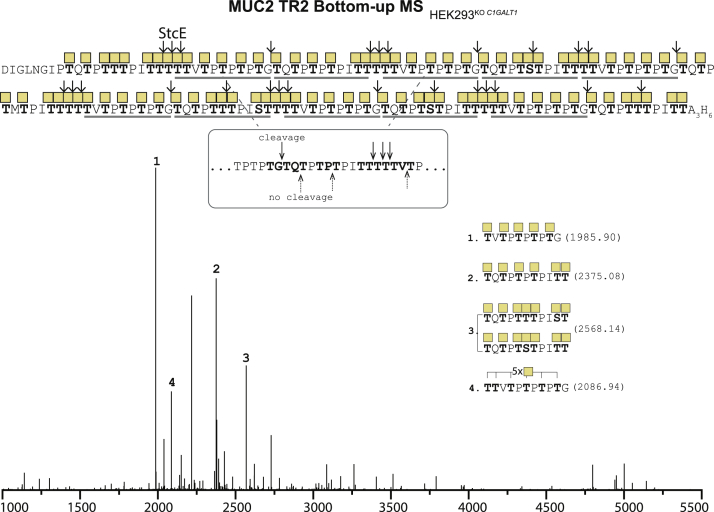
**Bottom–up analysis of O-glycodomains with Tn O-glycans using StcE.** Deconvoluted spectrum of bottom–up analysis of isolated MUC2 TR2 O-glycodomain from the reporter expressed in HEK293^KO^^*C1GALT1*^. Numbers assigned to each peak from one to four are given based on decreasing abundance. Experimental masses in dalton for each assigned peak are provided in Supporting File S1. Only peaks with intensity above 10% were assigned. HEK293, human embryonic kidney 293 cell line; TR, tandem repeat.

Glycomucinases may offer unique opportunities for use in bottom–up analysis of mucin TRs and other dense O-glycodomains (16, 71); however, they may digest TRs into fragments that are challenging to identify and/or challenging to place in sequence context, and detailed knowledge of the cleavage specificities of these enzymes is still limited (72). StcE is a zinc metalloprotease secreted from the human pathogenic enterohemorrhagic *Escherichia coli* (73). We previously showed that StcE efficiently cleaves the MUC2 and MUC5AC TR reporters (34), and here, we chose to analyze the products generated by StcE cleavage of the MUC2 TR2 reporter with Tn O-glycans (Figs. 6 and S7). We identified Tn-peptides covering most of the sequence and fully confirmed the high occupancy of O-glycosylation demonstrated by intact MS. This also revealed that StcE primarily cleaved in the TTT and TGT sequons and did not cleave in TPT, TQT, and TVT sequons. StcE was proposed to cleave in the S/TX↓S/T motif with an obligate O-glycan at P2 (16).

**Figure 7:**
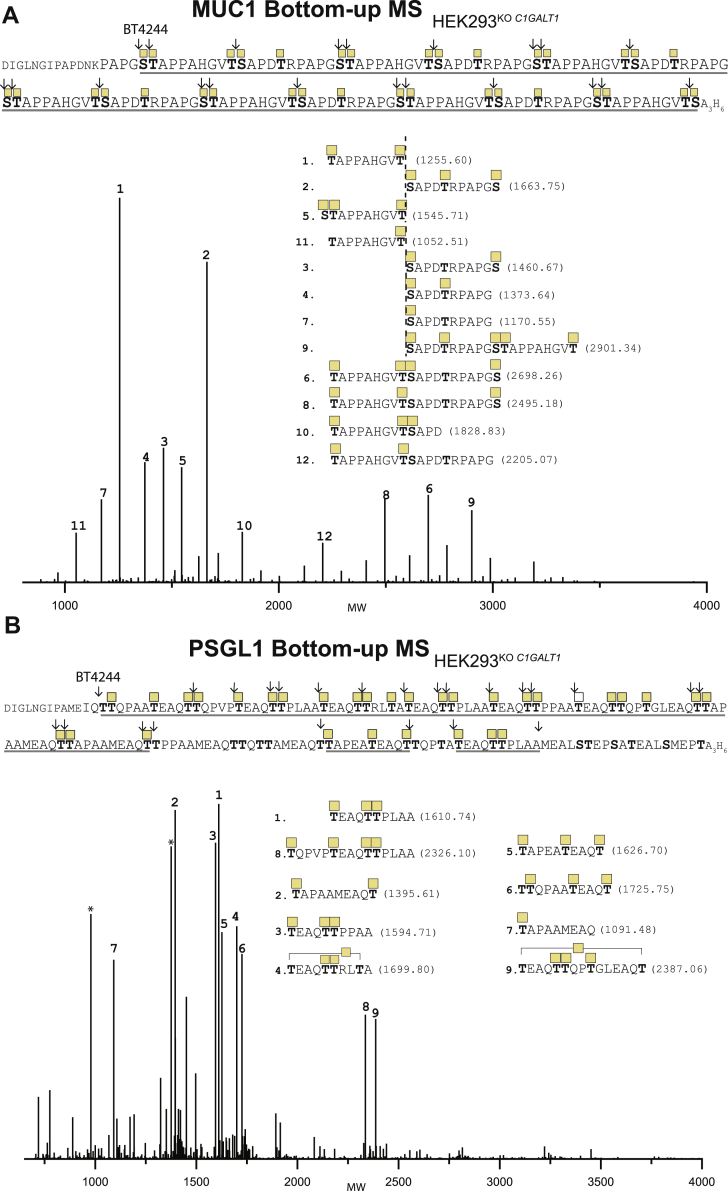
**Bottom–up analysis of O-glycodomains with Tn O-glycans using BT4244.***A*, deconvoluted spectrum of the bottom–up analysis of isolated MUC1 TR O-glycodomains from reporters expressed in HEK293^KO^^*C1GALT1*^. *B*, spectrum for PSGL-1. Numbers assigned to each peak are based on decreasing abundance. Experimental masses in dalton for each assigned peak are provided in Supporting File S1. Only peaks with intensity above 10% were assigned. HEK293, human embryonic kidney 293 cell line; PSGL-1, P-selectin-glycoprotein ligand 1; TR, tandem repeat.

We also used the glycomucinase BT4244 suggested to cleave N-terminal to Ser or Thr residues with Tn or T *O*-glycans attached (46, 47). BT4244 efficiently cleaved the Tn MUC1 reporter, and the predominant cleavage sites identified were in between the diad *O-*glycans in the GSTA and VTSA motifs, whereas no cleavage was found in the single PDTR O-glycosite (Figs. 7 and S8). One identified glycopeptide indicated cleavage N-terminal to Ser in the GSTA motif. BT4244 digestion of the PSGL-1 reporter revealed cleavage in between the diad O-glycans in the TT motif, as well as cleavage N-terminal to the single Thr in TEA, and occasionally cleavage N-terminal to the first Thr in QTT (Fig. 7*B*). While StcE and BT4244 digestion provided useful information on select mucin TRs as shown, it is also clear that their digestion patterns are complex and challenging to analyze, and careful selection of substrates is needed to obtain useful information.

### Applying proteases and glycomucinases to characterize OSM

**Figure 8:**
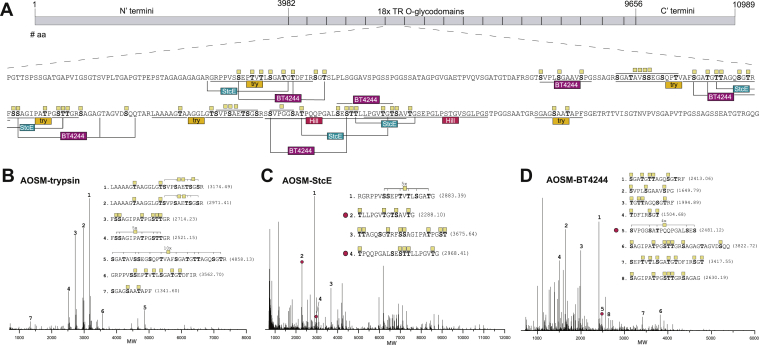
**Bottom–up analysis of OSM using trypsin, StcE, and BT4244.***A*, schematic depiction of the OSM mucin and a representative TR domain (333 amino acids) with a summary of identified glycopeptides indicated. Two peptides originally identified by Hill *et al.* (76, 77) by trypsin digestion of deglycosylated OSM are indicated (*magenta*), and a third peptide (STTQLPGVTGTSAVTGSEPGLPSTGVSGLPGS) is found in a variable repeat at the C-terminal junction not shown. *B*–*D*, deconvoluted spectra of bottom–up analysis of AOSM with homogenous Tn O-glycans digested with *trypsin* (*B*), StcE (*C*), and BT4244 (*D*). Numbers assigned to peaks are based on decreasing abundance. Experimental masses in dalton for each peak are provided in Supporting File S1. Only peaks with intensity above 10% were assigned. AOSM, asialo-OSM; OSM, ovine submaxillary mucin; TR, tandem repeat.

OSM was originally isolated and characterized by Bhargava and Gottschalk (74) and Tettamanti and Pigman (75), and three tryptic peptides were sequenced by Hill *et al.* (76, 77). The OSM gene to our knowledge has not been reported, but the peptide sequences could be identified by BLAST sequence search in a large gene (accession number: XP_027821751) with a putative 333 amino-acid Pro-Thr-Ser-rich TR predicted to represent the OSM gene (Fig. S9). OSM is a widely used isolated mucin that has rather homogenous STn (NeuAcα2–6GalNAcα1-O-Ser/Thr) O-glycans that after neuraminidase treatment is converted to asialo-OSM (AOSM) with Tn O-glycans (78, 79). We reproduced trypsin digestion of AOSM and identified five GalNAc-glycopeptides present in the putative TRs of the OSM gene (Fig. 8*B*). With StcE and BT4244 digestion, we identified additional GalNAc-glycopeptides (Fig. 8, *C* and *D*) that partly overlapped with the peptide sequences originally reported (76). We only identified two peptides outside the predicted TR region of OSM (Table 2). Interestingly, and in agreement with our studies of the mucin TR reporters, all identified glycopeptides had essentially complete O-glycan occupancy at all possible Ser/Thr residues.

**Table 2:** List of (glyco)peptides identified in the non-TR regions of OSM

Position in protein	Amino acid number in protein	Enzyme used	Sequence	Mass (Da)
N-terminal	933–946	Trypsin	AEDDFmSSQNILEK	1641.72
N-terminal	636–644	Trypsin	VSTLSSDYK	998.49
C-terminal	10,138–10,154	Trypsin	A**T**ISGS**S**H**T**EA**TT**LIAR	2730.29
C-terminal	10,009–10,019	Trypsin	LG**TT**VSTDGLK	1496.75
N/C-terminal	1763–1777/10,282–10,296	StcE	**T**AG**S**VG**TT**GLAGP**T**F	2351.07
C-terminal	10,642–10,669	StcE	**T**DFIRSG**T**RFPV**S**GGAV**S**PG**SS**PGG**SS**A	4261.92
N/C-terminal	1800–1811/10,319–10,330	BT4244	GS**T**GDTGFRAGG	1284.57
N-terminal	1779–1794	BT4244	S**S**GRI**S**G**ST**GV**S**V**S**AV	2668.23
C-terminal	10,778–10,789	BT4244	**S**AAAG**T**AAGGLG	1308.61

## Discussion

The densely O-glycosylated regions of mucin TRs and mucin-like domains remain an analytical challenge to overcome, and the approaches taken here provide strategies and advances towards overcoming this. First of all, our studies of recombinantly expressed reporters for mucin TRs and the isolated OSM mucin suggest that essentially all Ser/Thr residues available in mucin TRs are O-glycosylated and with near complete occupancy, with the notable exception of lower occupancy in a few transmembrane mucin TRs with distinct TR sequences. Furthermore, our preliminary studies of mucin TRs produced in cells with altered repertoire of expressed GALNTs confirm that the repertoire of GALNTs available is important, but given that only minor effects were observed with KO of *GALNT7*/*10*, these studies also indicate that the O-glycosylation of mucin TRs appears to be covered by considerable functional redundancies among GALNTs. Thus, changes in expression of individual GALNTs are probably less likely to affect the glycosylation of mucin TRs in contrary to what has been found for few select O-glycoproteins and more isolated glycosites (4, 51, 52, 53). These results suggest that mucins in contrast to the prevailing prediction may in fact be rather homogeneous molecules, at least with respect to the O-glycan occupancy. Our studies also provided further validation for the use of the glycoengineering and cell-based platform for production of mucin O-glycodomains with authentic glycosylation. This is especially important given the scarcity of natural human mucins and difficulties associated with their isolation, characterization, and consistency. Finally, we provided strategies for site-specific analysis of mucin TRs using rational selection and combination of proteases and glycoproteases, which opens up for more detailed studies of isolated mucins.

Mucins are generally considered highly heterogenous molecules, and variations may stem from the protein backbone, caused by genetic variations in numbers of TRs encoded by gene alleles, alternative splicing, and degradation (25, 26, 27, 80), but the main heterogeneity is predicted to arise from the non-template-driven O-glycosylation process resulting in great diversity in structures and positions of O-glycans (1, 81, 82). However, the notion of great heterogeneity in O-glycosylation of mucins may at least partly be ascribed to difficulties with isolation and characterization of natural mucins from homogeneous sources. The large size of most secreted mucins has largely prevented recombinant expression and analysis from more homogeneous cell sources. Studies of recombinant MUC1 have shown that O-glycosylation of the five sites in the MUC1 TRs is well occupied (83), although one study suggested that overexpression of GALNT4 was required for efficient glycosylation at the Ser in VTSA motifs (84). Moreover, in the original analysis of isolated OSM, quantitation of the total Ser/Thr and HexNAc content suggested that this native mucin had nearly complete O-glycan occupancy (77). Similarly, studies from Gerken *et al.* (17, 18, 19, 21) showed that TRs from porcine submaxillary mucin and canine submaxillary mucin were highly O-glycosylated. Most studies of O-glycans found on mucins use profiling of released O-glycans from isolated mucins derived from large and heterogenous cell and tissue sources. While these have shown great heterogeneity in the identified structures, these studies do not enable interpretation of the fidelity of O-glycosylation processes in a particular cell. Our studies of mucin TR reporters expressed in glycoengineered HEK293 cells suggest that these can in fact be produced with rather homogeneous O-glycan sites and structures (34).

Interestingly, we found that the core3 O-glycosylation pathway may interfere with the initiation process and occupancy of O-glycans at least in the case of the MUC1 TRs (Fig. 4). This is likely because of premature extension of initial GalNAc O-glycans by β3GlcNAc residues in competition with the follow-up GALNTs that use their C-terminal GalNAc-binding lectins for efficient glycosylation of substrate sites adjacent to initial attached O-glycans (56, 60, 69, 85). Importantly, the core3 trisaccharide structure (Galβ1–4GlcNAcβ1–3GalNAcα1-O-Ser/Thr) on the MUC1 TR reporter was also found to be assembled with high fidelity (Fig. 4).

So far, we have only been able to apply intact MS analysis with the more simple O-glycosylated TR reporters and mainly after removal of sialic acids, and thus, further improvements are needed. In addition, the issues of mass degeneracy preclude confident annotation of O-glycoforms of reporters expressing more than one type of O-glycan structures. However, intact measurements avoid known ionization bias of glycopeptides *versus* peptides, resulting in an underestimation of glycosite occupancy (45, 86). Therefore, a combined approach with intact MS for quantitative assessment of O-glycosylation landscape and bottom–up glycopeptide analysis for O-glycosite identification represents the most promising future avenue to unravel the complexities of mucin and mucin-like domain O-glycoproteins.

The bottom–up analytical strategies for mucin TRs performed here are not universal and ideally involve prior knowledge of the amino acid sequences to design the optimal combination of proteases and glycoproteases (Fig. 1). Traditional proteases are likely only useful for select transmembrane mucins and mucin-like domains as these have greater frequencies of amino acids in typical cleavage sites (Arg, Lys, Glu, and Asp), and it may be expected that adjacent O-glycans interfere with cleavage in variable ways depending on size and sialylation of the O-glycans. Conversely, the bacterial glycoproteases and glycomucinases, in particular StcE, offer a powerful way to cleave densely O-glycosylated domains (72), and here, further knowledge of cleavage sites and O-glycoform dependencies are needed. These enzymes may be dependent on particular O-glycan structures and/or glycopeptide motifs, for example, the OgpA (OpeRATOR) glycoprotease cleaves N-terminal to Ser/Thr residues with attached core1 O-glycans (87). Sialic acid and branching of the O-glycan inhibit cleavage. OgpA provides a valuable tool for general O-glycoproteomics strategies but perhaps is less informative when it comes to mucin TRs with high density of O-glycans leaving too small fragments. In contrast, the StcE glycomucinase was reported to cleave a specific sequence motif (S/TX↓S/T) with requirement of an O-glycan at P2 and option for an O-glycan at P1′, but with little influence by the actual structure of the O-glycan(s) (16). However, we found that StcE cleavage appears to be blocked by STn and core3 O-glycans (34), and here, we provided further insight into the peptide sequon for StcE finding that the consensus substrate motif may be refined to S/TX↓S/T, where X ≠ P/Q/V. This was particularly useful for bottom–up analysis of the MUC2 TRs that essentially only consist of the broader S/TX↓S/T motifs, since this enabled release of reasonable-sized glycopeptide fragments suitable for LC–MS/MS analysis (Fig. 6). We also used BT4244 reported to cleave N-terminally to Thr/Ser residues with Tn or T O-glycans (46) and preference for Tn (47). We found that BT4244 exhibits preference toward O-glycan diads (TS, TT, and ST). Mucin TR reporters are valuable tools to dissect the fine specificities of these important glycoproteases not only in terms of protein substrate motifs but also with respect to the role of O-glycan structures given the capabilities to produce distinct glycoforms with the glycoengineered cell–based expression platform.

We extended the bottom–up strategy to a native mucin to further explore our finding that the mucin TR reporters were glycosylated with essentially full occupancy. We selected the mucin OSM because it is known to have homogeneous STn O-glycans, and it is widely used for characterization of antibodies to the cancer-associated STn and Tn (after removal of sialic acids) O-glycans (78, 88, 89). A large fraction of the many monoclonal antibodies available to STn and Tn antigens were elicited with OSM and AOSM as the immunogen, and partially desialylated OSM was used in a clinical trial to stimulate immunity in cancer patients (90). Interestingly though, the gene and sequence of this mucin has not been described in the literature to our knowledge, but with three peptide sequences originally obtained by Hill *et al.* (76, 77), the putative OSM gene was identified by a BLAST search, and by use of proteases and glycomucinases, we could confirm the authenticity of the OSM gene (Figs. 8 and S9). Importantly, our analysis of AOSM revealed that this mucin being naturally glycosylated with STn O-glycans exhibited near complete occupancy, which confirms our findings not only of high occupancy of mucin TRs by analysis of the reporters but also that mucins can be produced with STn O-glycans in normal cells without interference with O-glycan occupancy by premature sialylation.

In summary, our studies provide deeper insights into O-glycosylation of mucins suggesting that these regions carry O-glycans at all potential Ser/Thr residues with near full stoichiometry. We provided further evidence for the cell-based mucin TR array platform as a valid model to study O-glycosylation of mucins and importantly to produce representative mucin fragments with custom-designed glycosylation. There are still considerable challenges in analyzing more complex glycoforms and natural mucins, but the intact MS and bottom–up analytic strategies developed should be of wider use and stimulate further progress.

## Experimental procedures

### Cell culture

HEK293 cells were cultured in Dulbecco's modified Eagle's medium (Sigma–Aldrich) supplemented with 10% heat-inactivated fetal bovine serum (Sigma–Aldrich) and 2 mM GlutaMAX (Gibco) in a humidified incubator at 37 °C and 5% CO_2_. A suspension HEK293 cell line was cultured in an orbital shaker in F17 medium (Gibco) supplemented with 0.1 Kolliphor P188 (Sigma–Aldrich) and 2% Glutamax. All glycoengineered isogenic HEK293 cells used in this study are available as part of the cell-based glycan array resource (35, 91).

### Production and purification of recombinant mucin TR reporters

The design and construction of the mucin TR reporters were previously reported (34, 36), and the full sequences used in this study are shown in Fig. S1. Glycoengineered HEK293 cell lines stably expressing secreted mucin reporters (34) were used for production by seeding cells at 0.25 × 10^6^ cells/ml and culturing for 5 days before harvesting. Secreted reporters were purified by nickel–nitrilotriacetic acid affinity (Qiagen) chromatography (pre-equilibration with 25 mM sodium phosphate, 0.5 M NaCl, 10 mM imidazole [pH 7.4] and eluted with addition of 250 mM imidazole). Purified reporters were analyzed by SDS-PAGE and quantified using a Pierce BCA Protein Assay Kit (Thermo Fisher Scientific).

### Isolation of mucin TR O-glycodomains

Purified mucin TR reporters were digested with Lys-C (Roche) (1:35 enzyme/substrate ratio) for 18 h at 37 °C in 50 mM ammonium bicarbonate buffer (pH 8.0). Reactions were heat inactivated at 98 °C for 10 min, and O-glycodomains were isolated by C4 HPLC (Aeris C4; 3.6 μm, 200 Å, 250 × 2.1 mm; Phenomenex) using 0% to 100% solvent B during 80 min (A: 0.1% TFA and B: 90% acetonitrile [ACN] in 0.1% TFA) with flow rate set at 0.2 ml/min. The fractions containing the O-glycodomain TR reporters were detected by VVA lectin ELISA. Collected fractions were freeze dried twice, and approximately, 1 μg was resuspended in 20 μl 0.1% formic acid (FA) and subjected to intact MS analysis. For MUC1-core3, after C4 HPLC purification, samples were desialylated with 40 mU *Clostridium perfringens* neuraminidase (Sigma–Aldrich) at 37 °C for 5 h in 50 mM sodium acetate buffer (pH 5.0) and subsequently purified by C18 HPLC (Aeris; 3.6 μm WIDEPORE XB-C18, 200 Å, 250 × 2.1 m; Phenomenex) using same chromatographic conditions as described previously.

### ELISA

MaxiSorp 96-well plates (Nunc) were coated with diluted HPLC fractions with concentrations from approximately 1 ng/μl. Plates were blocked with PLI-P buffer (PO_4_, Na/K, 1% Triton X-100, 1% bovine serum albumin, pH 7.4), incubated with 1 μg/ml biotinylated-lectin VVA (Vector Laboratories and Lectenz Bio) for 1 h at room temperature, followed by incubation with streptavidin-conjugated horseradish peroxidase (1:5000 dilution) (Dako) for 1 h, and treatment with TMB (3,3',5,5'-tetramethylbenzidine substrate) (Dako) and 0.5 M H_2_SO_4_ to stop reactions. Absorbance was read at 450 nm.

### Intact MS analysis

Intact MS analysis of mucin TRs was performed by EASY-nLC 1200 UHPLC (Thermo Scientific Scientific) interfaced *via* nanoSpray Flex ion source to an on Orbitrap Fusion/Lumos instrument (Thermo Fisher Scientific) using “high” mass range setting in *m/z* range 700 to 4000. Instrument was operated in “low pressure” mode to provide optimal detection of intact protein masses. MS parameter settings: spray voltage 2.2 kV and source fragmentation energy 35 V. All ions were detected in Orbitrap at a resolution of 7500 (at *m/z* 200). The number of microscans was set to 20. The nLC was operated in a single analytical column set up using PicoFrit Emitters (New Objectives; inner diameter of 75 mm) packed in-house with C4 phase (Dr Maisch, particle size of 3.0 mm; 16–20 cm column length) or C18 phase (Dr Maisch, particle size of 1.9 mm; column length of 16–20). Each sample was injected onto the column and eluted in gradients from 5 to 30% B in 25 min, from 30 to 100% B in 20 min, and 100% B for 15 min at 300 nl/min (solvent A, 100% H_2_O; solvent B, 80% ACN; both having 0.1% [v/v] FA).

### Bottom–up MS analysis

LC–MS/MS site-specific O-glycopeptide analysis of mucin TRs was performed on EASY-nLC 1000 UHPLC interfaced *via* nanoSpray Flex ion source to an Orbitrap Fusion MS or EASY-nLC 1000 UHPLC interfaced *via* New Objectives ion source to an OrbiTrap Fusion MS (Thermo Fisher Scientific). Briefly, the nLC was operated in a single analytical column set up using PicoFrit Emitters (New Objectives; inner diameterof 75 mm) packed in-house with Reprosil-Pure-AQ C18 phase (Dr Maisch; particle size of 1.9 mm, column length of 19–21 cm). Each sample was injected onto the column and eluted in gradients from 3 to 32% B for glycopeptides and 10 to 40% for released and labeled glycans in 45 min at 200 nl/min (solvent A, 100% H_2_O; solvent B, 100% ACN; both containing 0.1% [v/v] FA). A precursor MS1 scan (*m/z* 350–2000) of intact peptides was acquired in the Orbitrap at the nominal resolution setting of 120,000, followed by Orbitrap higher-energy collisional dissociation–MS2 and electron-transfer dissociation–MS2 at the nominal resolution setting of 60,000 of the five most abundant multiply charged precursors in the MS1 spectrum; a minimum MS1 signal threshold of 50,000 was used for triggering data-dependent fragmentation events. Targeted MS/MS analysis was performed by setting up a targeted MSn Scan Properties pane.

### Data analysis

For intact MS and site-specific bottom–up analysis, raw spectra were deconvoluted to zero charge by BioPharma Finder Software (Thermo Fisher Scientific) as previously described with minor modifications (34). Briefly, sliding windows method was used for chromatography and source spectra with target average spectrum width of 0.1 min. Xtract deconvolution algorithm was used for bottom–up data, and ReSpect deconvolution algorithm was used for intact MS data. Glycoproteoforms were annotated from zero-charge deconvoluted intact MS data by in-house written SysBioWare software (92) using average masses of hexose, HexNAc, and the known predicted mass of the mucin TR reporter sequences. For site-specific glycopeptide identification, the corresponding higher-energy collisional dissociation MS/MS and electron-transfer dissociation MS/MS were analyzed by Proteome Discoverer 2.2 software (Thermo Fisher Scientific).

### Mucin TR digestion for bottom–up MS analysis

For bottom–up analysis, C4 HPLC-purified O-glycodomains were digested with Asp-N (2× [1:35] ratio for 18 h at 37 °C, in 100 mM Tris–HCl buffer [pH 8.0]), Glu-C (1:10 ratio for 18 h at 37 °C in 50 mM ammonium bicarbonate buffer [pH 8.0]), or trypsin (1:8 ratio for 12 h at 37 °C in 50 mM ammonium bicarbonate buffer). Reactions were stopped with the addition of 1 μl concentrated TFA. Samples were desalted with Stage Tips (C18 sorbent-Empore 3M), freeze dried twice, and ∼1 μg was resuspended in 20 μl 0.1% FA for nLC–MS/MS. The MUC1-core3 after C4 HPLC purification was desialylated with 40 mU *C. perfringens* neuraminidase (Sigma–Aldrich) at 37 °C for 5 h in 50 mM sodium acetate buffer (pH 5.0) and subsequently purified by C18 HPLC.

Recombinant StcE and BT4244 enzymes were produced in *E. coli* as reported previously (34). BT4244 was produced with a codon-optimized sequence (residues 35–857) cloned into pet28 (kanamycin) (Twist Bioscience). Digestions were performed with secreted mucin reporters and BT4244 at an enzyme to substrate ratio of 1:50 for 3 h at 37 °C in 50 mM ammonium bicarbonate buffer (final volume of 100 μl) and StcE at an enzyme to substrate ratio of 1:50 for 1 h at 37 °C in H_2_O (final volume of 10 μl) followed by heat inactivation at 98 °C for 10 min. Peptides were purified by C18 HPLC. Fractions containing Tn-glycopeptides were detected by VVA lectin ELISA. The collected fractions were freeze dried twice, and approximately, 1 μg was resuspended in 20 μl 0.1% FA to be further analyzed by nLC–MS/MS.

## Data availability

All data generated or analyzed during this study are included in this article and supporting information files.

The MS data have been deposited to the ProteomeXchange Consortium *via* the PRIDE (93) partner repository with the dataset identifier PXD029885.

## Supporting information

This article contains supporting information. Supporting information includes Supporting Figs. (S1–S9) and one excel file (Supporting File S1) (34, 76, 77).

## Conflict of interest

The University of Copenhagen has filed a patent application on the cell-based display platform. GlycoDisplay Aps, Copenhagen, Denmark, has obtained a license to the field of the patent application. Y. N. and H. C. are cofounders of GlycoDisplay Aps and hold ownerships in the company. All the other authors declare that they have no conflicts of interest with the contents of this article.
