# Supplementary material for: Exploring the glycosylation of mucins by use of O-glycodomain reporters recombinantly expressed in glycoengineered HEK293 cells
Source: J Biol Chem. 2022 Mar 2;298(4):101784. doi: 10.1016/j.jbc.2022.101784 (PMC8980628; doi:10.1016/j.jbc.2022.101784)
Supplement: Supplemental Figures S1–S9 [file mmc2.pdf]

SUPPORTING INFORMATION

Exploring the glycosylation of mucins by use of O-glycodomain reporters recombinantly expressed in glycoengineered HEK293 cells.

Andriana Konstantinidi[1], Rebecca Nason[1], Tomislav Čaval[1], Lingbo Sun[1,^], Daniel M. Sørensen[1], Sanae Furukawa[1], Zilu Ye[1#], Renaud Vincentelli[2], Yoshiki Narimatsu[1,3], Sergey Y. Vakhrushev[1*], Henrik Clausen[1*]

[1] Copenhagen Center for Glycomics, Departments of Cellular and Molecular Medicine, Faculty of Health Sciences, University of Copenhagen, Blegdamsvej 3, Copenhagen, Denmark

[2] Architecture et Fonction des Macromolécules Biologiques, CNRS, Aix-Marseille Université, Marseille, France

[3] GlycoDisplay ApS, Copenhagen, Denmark

* Correspondence: hclau@sund.ku.dk and seva@sund.ku.dk

Table of Contents

Figure S1. **Design of the human mucin TR reporters with schematic presentation of the imperfect TR amino acid sequences selected from the human mucin genes.**

Figure S2. Intact MS analysis of the MUC22 TR reporter containing an N-glycan.

Figure S3. Intact MS analysis of O-glycodomains isolated from mucin TR reporters with altered O-glycan occupancy.

Figure S4. Bottom-up analysis of the MUC1 TR reporter with non-altered O-glycan occupancy.

Figure S5. HPLC isolation of the MUC1 reporter expressed in HEK293$^{\mathrm{KO}\ \mathit{COSMC},\ \mathrm{KI\ B3GNT6}}$ cells.

Figure S6. Bottom-up analysis of the MUC20 and MUC21 TR reporters.

Figure S7. Analysis of StcE digestion of the MUC2 TR2 reporter.

Figure S8. Analysis of BT4244 digestion of the MUC1 TR reporter.

Figure S9. The full amino acid sequence of the OSM mucin deduced from Accession number: XP_027821751.

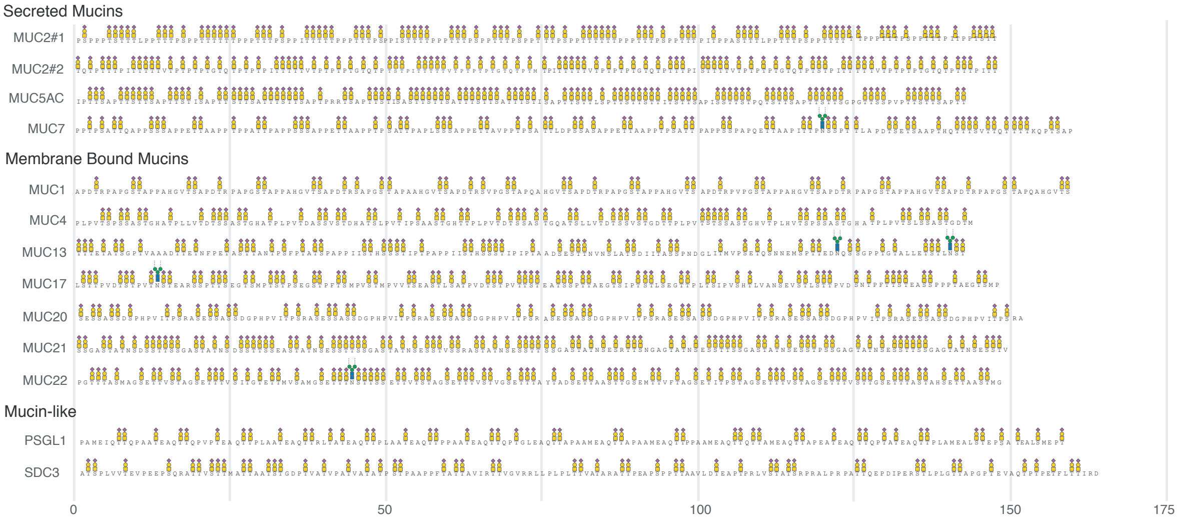

Figure S1. **Design of the human mucin TR reporters with schematic presentation of the imperfect TR amino acid sequences selected from the human mucin genes.** All Ser/Thr residues are highlighted as potential O-glycosites by glycan symbols (mSTa O-glycans shown for simplicity) to illustrate the characteristic patterning generated with all Ser/Thr residues O-glycosylated. An indicative N-glycan is attached to asparagine residues at N-glycosylation consensus sites (NXS/T). Note that SDC3 does not contain TRs, and the TRs of MUC13 are highly variable.

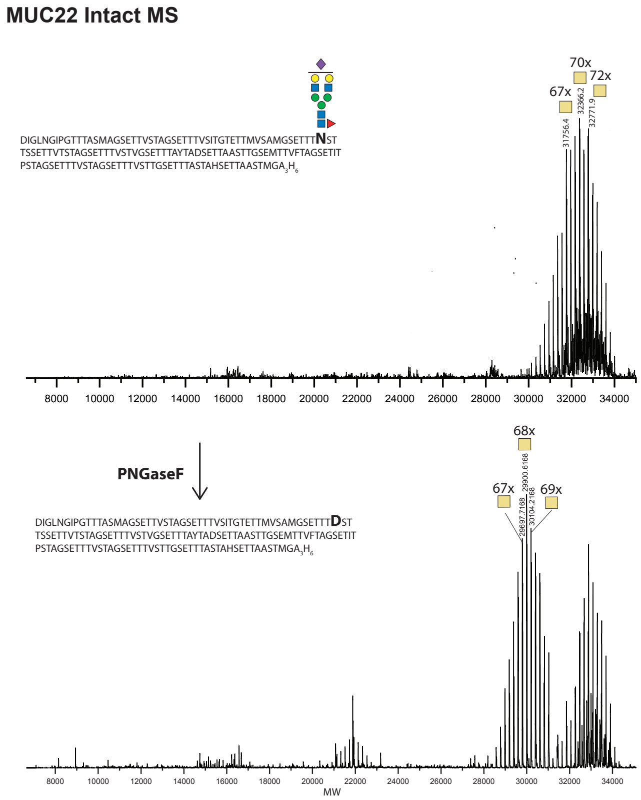

Figure S2. **Intact MS analysis of the MUC22 TR reporter containing an N-glycan.** The upper mucin TR reporter was expressed in HEK293$^{KO\ COSMC}$ and the lower MUC22 mucin TR reporter in HEK293$^{KO\ C1GALT1}$ cells, both with GalNAcα1-O-Ser/Thr (Tn) O-glycans. The purified reporters were digested with trypsin and the TR O-glycodomains were isolated by C4 HPLC. The isolated O-glycodomain was treated with PNGase F (lower panel). The 3 most abundant masses are annotated with predicted number of attached HexNAc residues. Relative abundances, deconvoluted masses, annotation and theoretical masses of all peaks above 5% intensity are given in **Supporting File 1**.

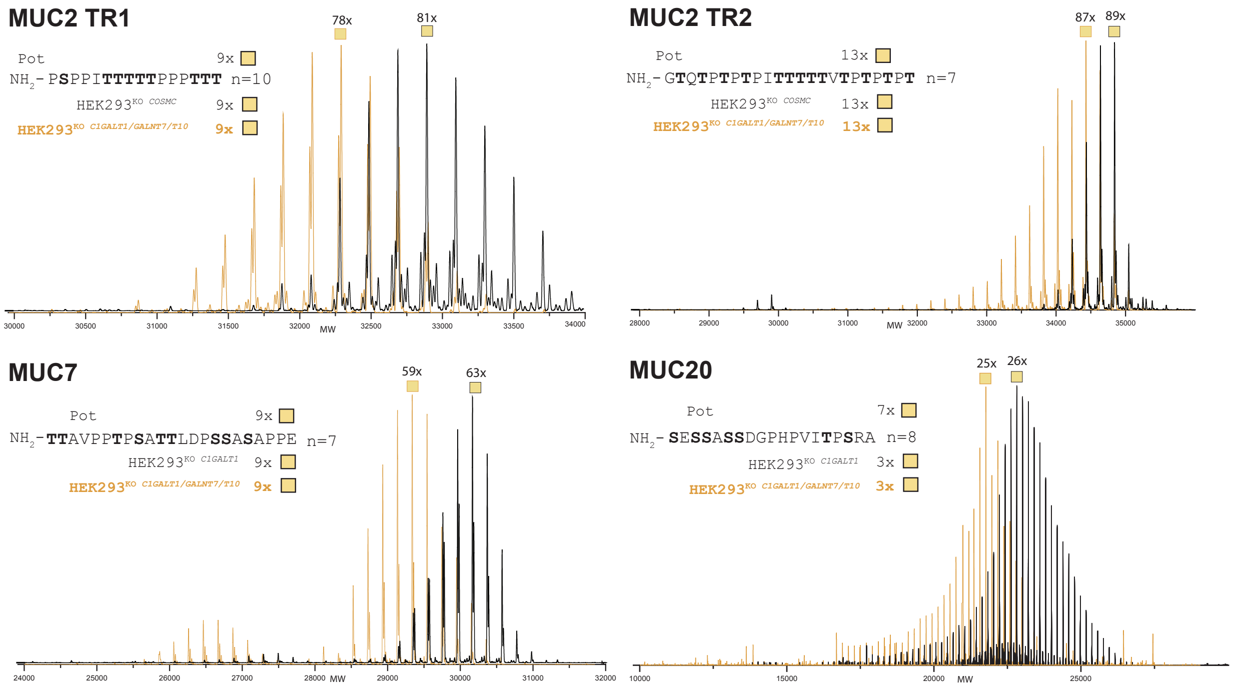

Figure S3. Intact MS analysis of O-glycodomains isolated from mucin TR reporters with non-altered O-glycan occupancy. Overlay of deconvoluted intact mass spectra of mucin TR reporters produced either in HEK293$^{KO\ C1GALT1}$ or in HEK293$^{KO\ COSMC}$ (black contour) and in HEK293$^{KO\ C1GALT1,GALNT7/10}$ (orange contour). The most abundant masses are annotated with predicted number of HexNAc residues. A representative TR sequence is shown with indicated total potential glycosylated Ser/Thr residues (Pot) and the experimentally determined average number of HexNAcs found per each TR domain (HEK293$^{KO\ C1GALT1/COSMC}$, HEK293$^{KO\ C1GALT1/GALNT7/T10}$). Relative abundances, deconvoluted masses, annotation and theoretical masses of all peaks above 5% intensity are given in **Supporting File 1.** For MUC2 TR1, MUC2 TR2 and MUC7, we used the same raw files from previous work (1), but we modified the deconvolution parameters (see Data Analysis in Experimental Procedures section).

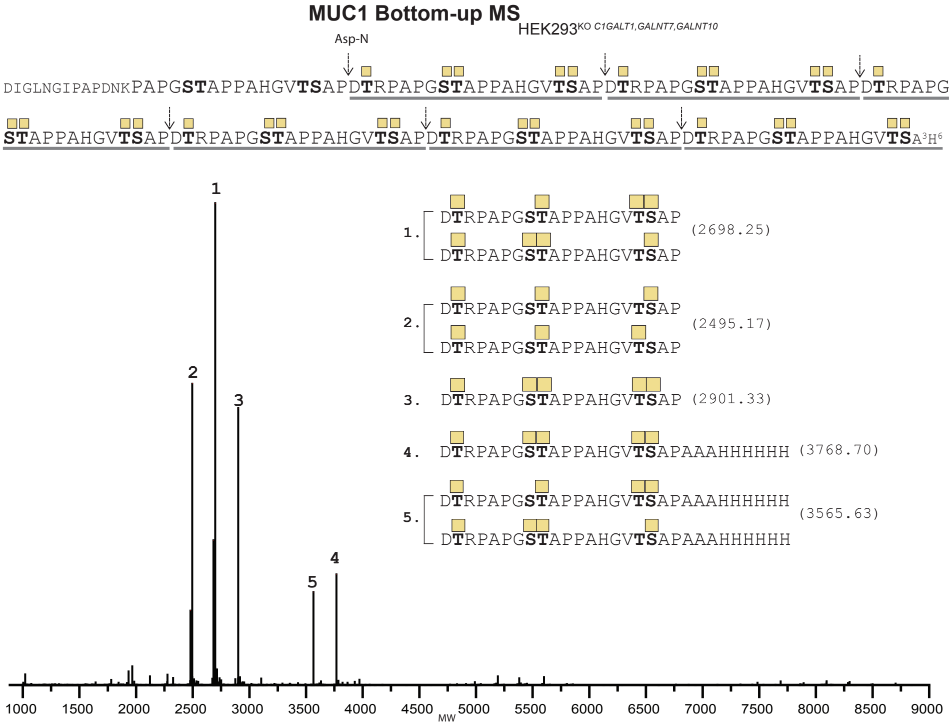

Figure S4. **Bottom-up analysis of the MUC1 TR reporter with altered O-glycan occupancy.** Deconvoluted spectrum of the bottom-up analysis of the MUC1 TR O-glycodomain expressed in HEK293 $^{KO\ C1GALT1,GALNT7,GALNT10}$. The full sequence of the MUC1 TR O-glycodomain is provided as a visual guide for observed fragments with arrows indicating Asp-N cleavage sites. Bold letters in the full sequences represent unambiguously annotated glycosites validated by MS/MS and yellow squares for GalNAc indicate glycosylated sites. Underlining in the full sequence represents the identified sequences. Bold letters in the peptides represent potential O-glycosites. The numbers assigned to each peak from 1 to 5 are given based on decreasing abundance. Relative abundances, deconvoluted masses, annotation and theoretical masses of all peaks above 5% intensity are given in **Supporting File 1**.

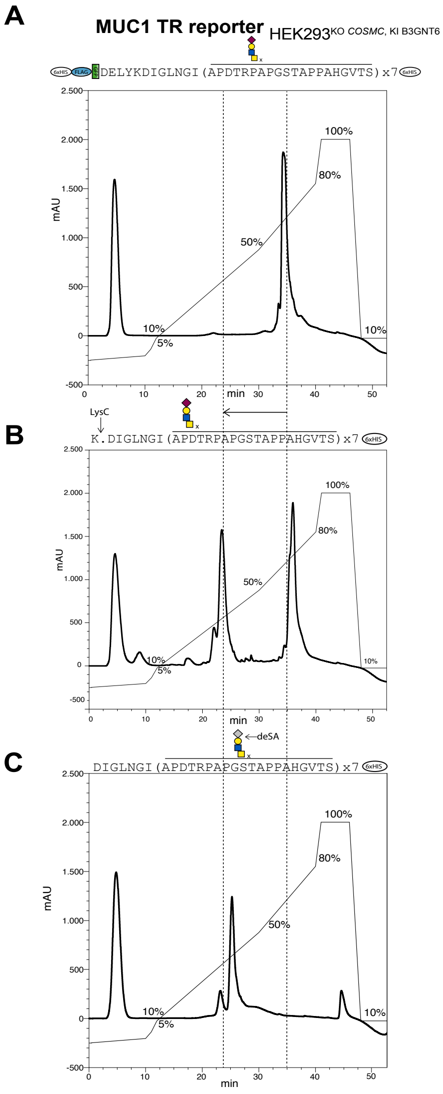

Figure S5. **HPLC isolation of the MUC1 reporter expressed in HEK293$^{KO\ COSMC,\ KI\ B3GNT6}$ cells.** *A,* C4 RP-HPLC elution trace (210 nm) of the intact MUC1 core3 TR reporter after Ni-NTA purification. *B,* C4 RP-HPLC elution trace after Lys-C digestion. *C,* C18 RP-HPLC elution trace of the C4 HPLC isolated O-glycodomain.

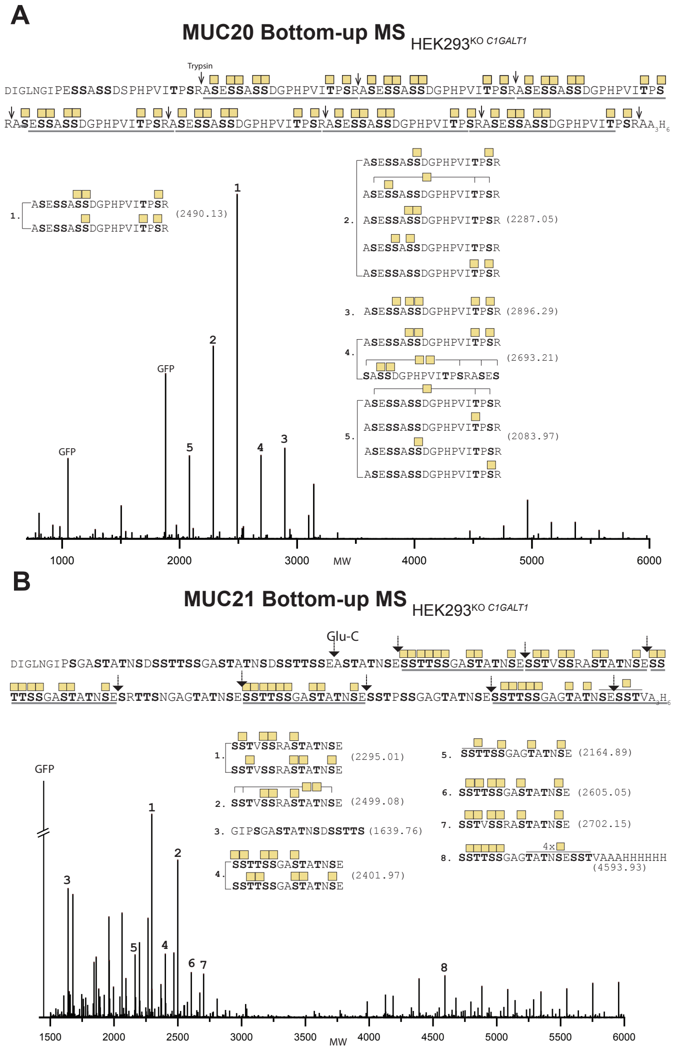

Figure S6. Bottom-up analysis of the MUC20 and MUC21 TR reporters. *A*, deconvoluted spectrum of MUC20. *B*, deconvoluted spectrum of MUC21. The full sequences of MUC20 and MUC21 TR O-glycodomains are provided as a visual guide for observed fragments with arrows indicating trypsin and Glu-C cleavage sites. Bold letters in the full sequences represent unambiguously annotated glycosites validated by MS/MS and yellow squares for GalNAc indicate glycosylated sites. Underlining represents the identified sequences. Bold letters represent potential O-glycosites. Connecting lines from yellow squares above peptides denote potential positions for ambiguously identified HexNAcs. Numbers assigned peaks from 1 to 5 (*A*) and from 1 to 8 (*B*) are given based on decreasing abundance. Relative abundances, deconvoluted masses, annotation and theoretical masses of all peaks above 5% intensity are given in **Supporting File 1**.

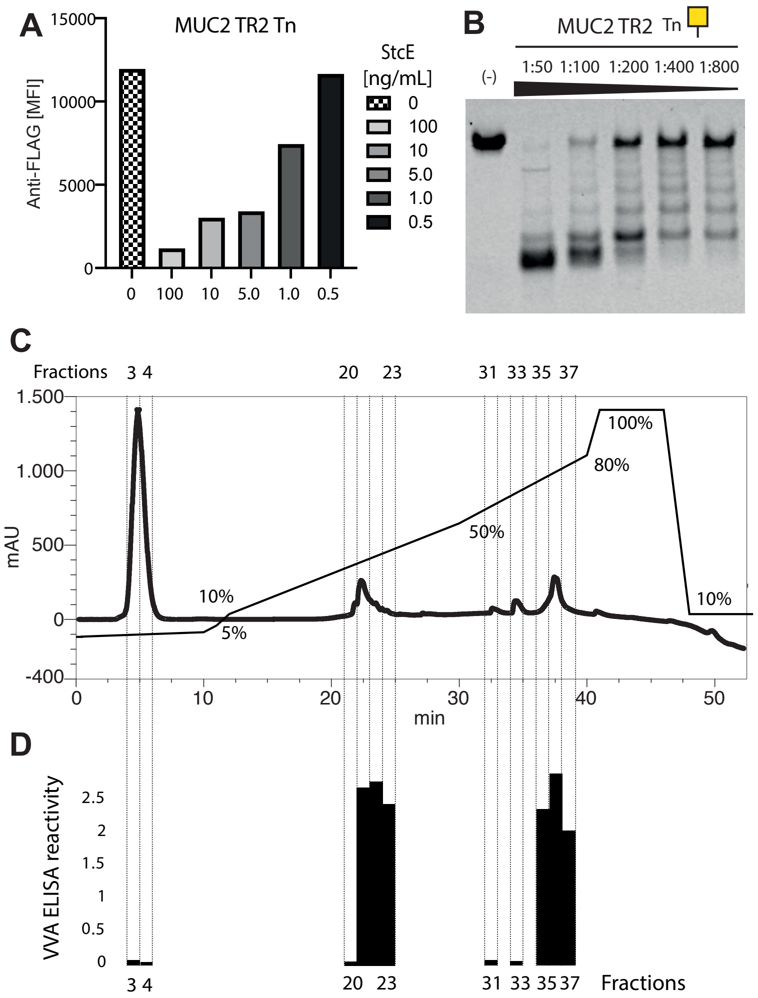

Figure S7. **Analysis of StcE digestion of the MUC2 TR2 reporter.** *A,* flow cytometry analysis of StcE cleavage (0-100 ng/ml dose titration) of MUC2 TR2 membrane bound reporters transiently expressed on HEK293$^{KO\ C1GALT1}$ cells (Tn glycoform) and detected by anti-FLAG antibody binding, as previously described (1). *B*, SDS-PAGE analysis of StcE digestion with dose titration of enzyme to substrate ratio (1:50 to 1:800) of the secreted purified MUC2 TR2 reporter expressed in HEK293$^{KO\ C1GALT1}$ cells (Krypton Fluorescent Protein Staining, Thermo Fisher Scientrific). *C*, C18 RP-HPLC 210 nm trace of the secreted purified MUC2 TR2 reporter expressed in HEK293$^{KO\ C1GALT1}$ digested by StcE (1:50) using gradient 0% to 100% (0-90% ACN in 0.1%TFA). *D*, VVA lectin ELISA assay of collected HPLC fractions from *C* detecting fractions containing Tn-MUC2 glycopeptides (21-23) and the undigested Tn-MUC2 TR reporter (35-37).

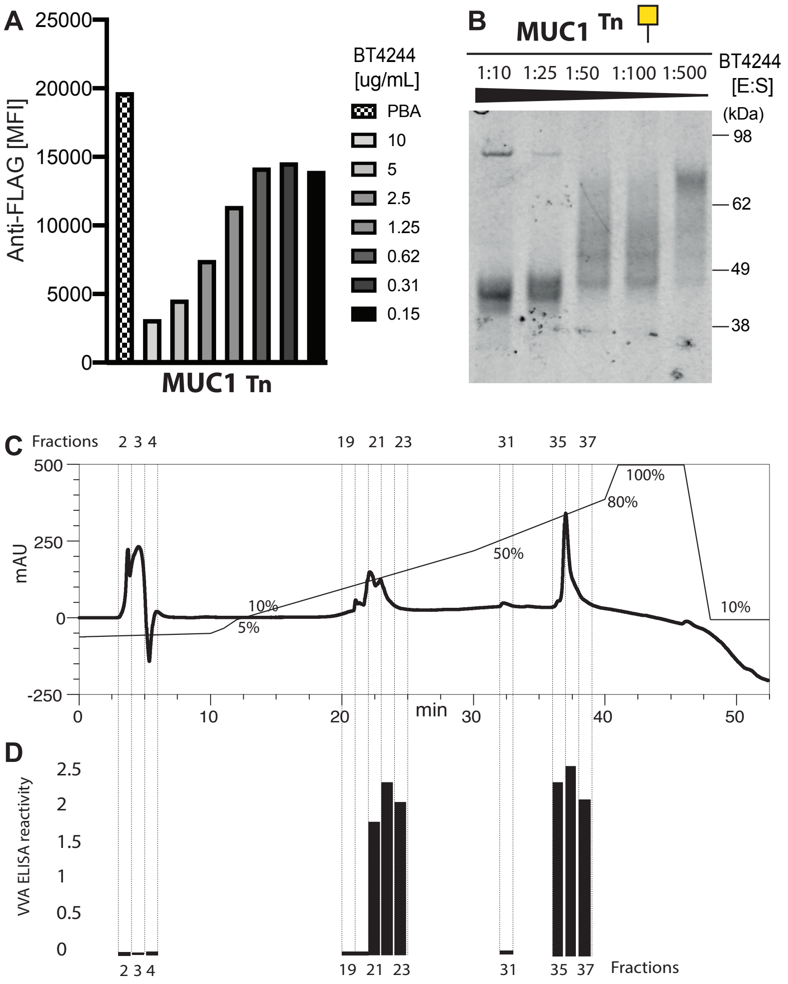

Figure S8. **Analysis of BT4244 digestion of the MUC1 TR reporter.** *A*, flow cytometry analysis of BT4244 digestion (0-10 µg/ml dose titration) of the MUC1 TR membrane reporter expressed transiently on HEK293$^{KO\ \mathit{C1GALT1}}$ cells (Tn glycoform) and detected by anti-FLAG antibody binding. *B*, SDS-PAGE analysis of BT4244 digestion with dose titration of enzyme to substrate ratio (1:10 to 1:500) of the secreted purified MUC1 TR reporter expressed in HEK293$^{KO\ \mathit{C1GALT1}}$ cells (Krypton Fluorescent Protein Staining, Thermo Fisher Scientific). *C,* C18 RP-HPLC 210 nm trace of MUC1 TR reporter expressed in HEK293$^{KO\ \mathit{C1GALT1}}$ cells digested with BT4244 (1:50) with gradient 0% to 100% solvent B (0-90% ACN in 0.1%TFA). *D,* VVA lectin ELISA of collected fractions from *C* containing Tn-MUC1 glycopeptides (21-23) and the undigested reporter (35-37).

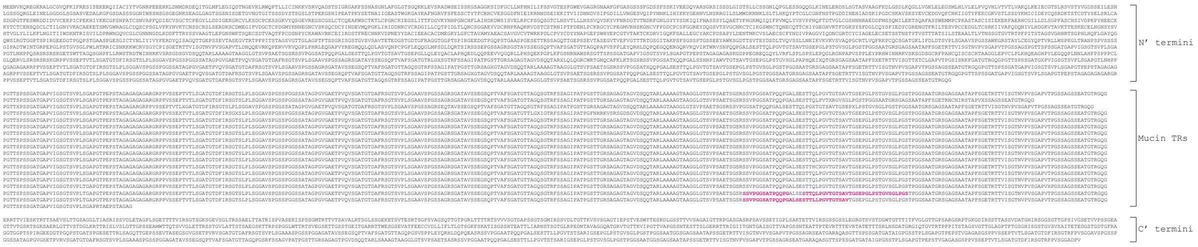

Figure S9. **The full amino acid sequence of the OSM mucin deduced from Accession number: XP_027821751**. Sequences in Magenda letters represent the peptides originally identified by Hill and colleagues (2, 3), which were used to identify the OSM gene by BLAST analysis of the porcine genome. The TRs of OSM are predicted to be approximately 333 amino acids and the 18 TRs are relatively conserved in sequence and length.

References

1. Nason, R., Büll, C., Konstantinidi, A., Sun, L., Ye, Z., Halim, A., Du, W., Sørensen, D. M., Durbesson, F., Furukawa, S., Mandel, U., Joshi, H. J., Dworkin, L. A., Hansen, L., David, L., Iverson, T. M., Bensing, B. A., Sullam, P. M., Varki, A., Vries, E. de, de Haan, C. A. M., Vincentelli, R., Henrissat, B., Vakhrushev, S. Y., Clausen, H., and Narimatsu, Y. (2021) Display of the human mucinome with defined O-glycans by gene engineered cells. *Nat. Commun.* **12**, 1–16
2. Hill, H. D., Schwyzer, M., Steinman, H., and Hill, R. L. (1977) Ovine submaxillary mucin. Primary structure and peptide substrates of UDP N acetylgalactosamine mucin transferase. *J. Biol. Chem.* **252**, 3799–3804
3. Hill, H. D., Reynolds, J. A., and Hill, R. L. (1977) Purification, composition, molecular weight, and subunit structure of ovine submaxillary mucin. *J. Biol. Chem.* **252**, 3791–3798
